# Provider payment approaches and incentives to implement screening

**DOI:** 10.1186/1940-0640-8-S1-A34

**Published:** 2013-09-04

**Authors:** Constance M Horgan, Deborah W Garnick, Maureen T Stewart, Dominic Hodgkin, Sharon Reif, Amity Quinn, Mary F Brolin

**Affiliations:** 1 Institute for Behavioral Health, Heller School for Social Policy and Management, Brandeis University, Waltham, MA, USA

## 

Screening in primary care is widely recommended (e.g. U.S. Preventive Services Task Force and UK National Institute for Health and Care Excellence), and is increasingly targeted for improvement. As health care payers try to influence the implementation and delivery of alcohol screening and brief intervention (SBI), some are using provider payment approaches. These include specific reimbursement for conducting screening, other financial incentives and linking payment with performance measures. We present 2010 data from a nationally representative survey of private US health plans and US health plan claims data (private health plans and Medicare), supplemented by literature review. Results indicate, among private health plan products in the US, 72.6% report allowing primary care physicians to bill for alcohol SBI, yet claims data analysis found only 0.01% of individuals in private plans or Medicare had an SBI procedure code on a medical claim in 2010. As of April 2013 the UK National Health Service requires that primary care practices conduct screening of individuals 16 and over and reimburses for SBI. In the US, although financial incentives for providers are becoming more common in private health plans (used by 31.6% for any condition), only 6% of health plans provided financial incentives for primary care screening for alcohol and drug use problems. Provider payment policies may be tied to performance measures to improve the quality of care. Alcohol screening is one of more than 300 performance measures selected for inclusion in the US Centers for Medicare and Medicaid Services (CMS) Physician Quality Reporting System. Despite this recognition, CMS analyses indicate the alcohol screening measure was not among the top five measures chosen by primary care providers. Although alcohol SBI is beneficial, it is not as widely implemented as it could be. Favorable payment policies are necessary, but not sufficient to encourage implementation of SBI.

